# Methodologies in Orthodontic Pain Management: A Review

**DOI:** 10.2174/1874210601711010492

**Published:** 2017-08-31

**Authors:** Asra Sabir Hussain, Meteib Joraib Al Toubity, Wael Y. Elias

**Affiliations:** 1 Private Dental Practice, Jeddah, Saudi Arabia; 2 Ministry of Health Hospital, Taif, Saudi Arabia; 3 Oral Diagnostic Science Department, King Abdul-Aziz University, Faculty of Dentistry, Jeddah, KSA.

**Keywords:** Orthodontics, Pain management, Pain control, Analgesics, Orthodontic pain, Discomfort

## Abstract

**Introduction:**

Patients experience pain and discomfort during active orthodontic treatment with fixed appliances. Pain is considered a subjective response to noxious stimuli. It can vary from person to person and is influenced by certain factors such as age, gender, previous pain experiences, stress or anxiety, and type of appliance.

**Objective:**

The objective of this literature review was to discuss conventional versus recently introduced treatment modalities used in pain management for orthodontic patients.

**Discussion:**

According to the reviewed articles, both pharmacological and non-pharmacological methods were introduced in orthodontic pain management. However, orthodontists must use their best professional judgment to assess each case individually and select an appropriate treatment modality based on pain threshold level of an individual.

**Conclusion:**

Nevertheless, further investigations are required in this field.

## 1 INTRODUCTION

Pain and discomfort are a common complication during active orthodontic treatment with fixed appliances. Pain relatively starts within four hours, increases over the next 24 hours, and decreases within seven days after initial bonding and placement of separators. The main cause of pain and discomfort in orthodontic treatment is due to multiple factors such as pressure, ischemia, inflammation, and edema related to tooth movement. The most common method of measuring pain intensity is the numerical rating scale (NRS) and visual analogue scale (VAS). Pharmacological and non-pharmacological methods can be relatively used in orthodontic pain management [1]. Moreover, discomfort associated with the fixed orthodontic appliances often exerts considerable impact on the quality of life of orthodontic patients. The determinant factors that cause discomforts usually are oral hygiene, speech impairment, difficulty in eating, tooth mobility, impairment of taste, halitosis and gingival bleeding [2].

Pain intensity can also be affected by the type of appliance. Patients with fixed appliances report significantly greater pain intensity than those with removable ones [3]. Invisalign aligners, besides other proposed benefits, have been proven to offer less pain compared to the fixed orthodontic appliances during the initial stages of treatment [4].

A simple and non-invasive preoperative sensory technique of using a cold pressor test aids in predicting the risk of developing pain in patients undergoing orthodontic treatment. Self-reported pain was reduced after archwire placements as the individual's pain tolerance increased. This experiment might help highlight an individual’s variations and preferred method of pain management [5].

Nevertheless, some patients might suffer from underlying orofacial pain conditions. A multidisciplinary approach is required in the management of orofacial pain disorders [6]. Therefore, orthodontists must collaborate with the orofacial specialists when encountering these patients in their practice.

The objective of this review was to discuss conventional versus recently introduced treatment modalities used in pain management for orthodontic patients.

## 2 LITERATURE REVIEW/ DISCUSSION

### 2.1 Pharmacological Management

Several studies discussed the importance of pharmacological management of pain during orthodontic treatment. Angelopoulou *et al.* conducted a meta-analysis to evaluate the effect of non-steroidal anti-inflammatory drugs (NSAIDs) in managing pain. Of the 1,127 studies, seven were included. No statistically significant difference was found between ibuprofen and paracetamol (acetaminophen) administration in relieving pain. It was evident from the literature that ibuprofen lowered pain at two hours and six hours after intervention but not at 24 hours when pain peaks. To some extent, ibuprofen relieves pain in the initial stage of treatment [7]. A review by Possi and Gallelli described the role of ibuprofen in orthodontic pain management and the authors concluded that ibuprofen 400 mg, if taken one hour before, three hours after, and seven hours postoperatively, significantly reduced pain. However, additional doses of ibuprofen were suggested after the seven hour dose to maintain the benefits of the medication when pain peaks at 24 hours after intervention. Hence, it was considered a safe and effective drug compared to other NSAIDs and also had comparatively fewer adverse effects. Some clinicians supported that NSAIDs had no impact on tooth movements as they were administered in lower doses and for a shorter duration. Therefore, in healthy individuals they are cleared by the body before tooth movement occurs. On the contrary, other authors declared a delay in orthodontic tooth movements as a result of NSAID intake [8].

A report based on a literature review states that NSAIDs impede tooth movement and also increase the risk of root resorption. Therefore, paracetamol (acetaminophen) was considered the safest NSAID that had no influence on the range of tooth movements as well as root resorption and other adverse effects that might occur within the oral cavity [9]. However, much of what has been published on this subject is still controversial.

On the other hand, preoperative analgesics were found to be effective in reducing pain and discomfort after intervention in patients with dental anxiety and fear [10]. Another study supported that premedication with piroxicam 20 mg significantly decreased pain in comparison to ibuprofen 400 mg. The administration of piroxicam 20 mg one hour before separator placement was recommended [11]. A study was conducted on 51 participants to assess the effects of a single dose of anti-inflammatory medication to preemptively treat orthodontic pain. The authors concluded that the use of a single dose of medication (lumiracoxib 400 mg) was not promising in preemptive orthodontic pain management. Nevertheless, pain severity was reportedly lower in individuals receiving the placebo group [12].

A study compared the preoperative administration of conventional NSAIDs (ibuprofen and acetaminophen) to meloxicam. Meloxicam administered at a dose of 7.5 mg was as effective as acetaminophen 650 mg and ibuprofen 400 mg in controlling orthodontic pain. Acetaminophen was declared the treatment of choice, as the drug had no gastrointestinal tract (GIT) toxicity and did not impede tooth movements. Nevertheless, meloxicam, which has the least GIT toxicity, can be an alternative option for patients in whom NSAIDs are contraindicated [13].

In a recent study by M. Abu Al-Melh and Andersson [14], the effect of lidocaine/prilocaine topical anesthetic was investigated on pain and discomfort attributed to the insertion of elastomeric separators. Hence, the overall mean discomfort/pain score was found to be significantly lower (*p*<0.001) with the topical anesthetic as compared to the placebo group. Therefore, this methodology might be beneficial for patients with a low pain threshold as it could potentially relieve pain and discomfort after the placement of orthodontic elastomeric separators.

### 2.2 Non-Pharmacological Management

Other treatment modalities in orthodontic treatment include chewing gum and biting on wafers. A study was conducted on 57 orthodontic patients to determine whether the use of chewing gum reduced the impact and pain caused by fixed orthodontic appliances. The difference between the median total impact score at 24 hours was 16 (*p*=0.031), and the median VAS between the two groups was 25 mm at 24 hours (*p*=0.038). Hence, the authors concluded that chewing gum significantly decreased both the impact and pain from fixed orthodontic appliances. There was no evidence that chewing gum increased the incidence of appliance breakages [15], but this is a controversial matter because patients are typically advised to avoid chewing gum during orthodontic treatment. On the other hand, a study designed to assess the pain response between the two groups found that pain management in the bite wafers group was not inferior to that of the over-the-counter (OTC) group (*p*>0.39). Therefore, BW (bite wafers) were also considered as effective as OTC analgesics for pain control in adolescents [16].

A recent study was initiated to investigate the effect of sugar-free chewing gum versus ibuprofen on reported pain in orthodontic patients. Patients were divided into two groups: a control group (ibuprofen only) and an experimental group (allowed to use ibuprofen if required along with the chewing gum). In conclusion, sugar-free chewing gum may reduce the level of ibuprofen use in the experimental group. However, it has no clinical/statistical significant effect on bond failures [17].

A research demonstrated that the use of systemic acupuncture preoperatively by qualified professionals can reduce pain in both male and female patients. Hence, it was considered a safe method of pain control [18].

Moreover, studies have emphasized that psychological perspective and behavioral management might help in preventing pain in orthodontic patients. Studies focused on the idea of establishing good orthodontist-patient communication. Pain can be prevented if orthodontists explain and guide their patients about expected pain during treatment [19, 20]. In the same line, cognitive behavioral therapy was effective in pain control during the initial stages of orthodontic therapy [21]. Moreover, patients who had a higher personal perception of their pain intensity and demonstrated attitudes characteristic for internal control orientation appeared to adapt faster and experience less pain and discomfort during active treatment [22]. Another study was designed to assess patients’ anticipation of pain and its side effects before orthodontic therapy and self-reported pain after archwire placement. The authors concluded that patients underestimated the dietary changes they would have to make due to post-archwire placement, and those who anticipated a greater effect of pain reported experiencing a higher intensity of pain and a higher negative impact of pain on their lifestyle [23].

However, a recent database search revealed that the quality of evidence for non-pharmacological interventions to manage orthodontic pain was very low. Further prospective research with long follow-up is required [24].

Low-level laser therapy (LLLT) had been introduced earlier as another treatment modality for orthodontic pain control. Besides its analgesic effect, LLLT enhances tissue recovery and accelerates tooth movement. Several studies have discussed the proposed benefits of this treatment modality. A meta-analysis was conducted, and out of 186 results, only 14 randomized clinical trials (RCTs) with a total of 659 participants met the inclusion criteria. The results showed that diode LLLT significantly reduced pain by 39% compared to the placebo groups (*p* = 0.02) [25]. However, studies were not able to provide sufficient evidence to support the effectiveness of LLLTs. Furthermore, research with better designs and appropriate samples should be conducted to recommend the use of LLLT [26, 27].

Single-dose, helium-neon laser therapy was found to be effective in reducing orthodontic pain in patients with maxillary canine retractions. The therapy contributed to a 12.1% pain reduction compared to the placebo group. But the study had its own limitations, and no previous studies had investigated the effectiveness of helium-neon laser therapy versus other laser types [28].

A recent systematic review demonstrated that LLLT might improve orthodontic treatment by accelerating tooth movements and modulating acute pain, as well as preventing relapse. The quality of scientific evidence that supported LLLT use in modulating acute pain was low. Further research with robust study designs and conformity of laser methods will be required to reveal the worth of LLLTs in modern times as a routine method of orthodontic pain control [29]. Bayani and colleagues designed a study evaluating the efficacy of ibuprofen, bite wafer and low power red and infrared lasers in orthodontic pain management. The authors concluded that a single session from a low power infrared laser was the most effective and suitable methodology for pain relief in orthodontic patients following archwire placement. However, on the contrary, low power red laser irradiation was not suggested. Chewing on bite wafers could be used as an alternative option to ibuprofen [30].

Analgesics and laser irradiation remained effective in the management of orthodontic pain at its peak intensity among all used interventions. However, further research is required to improve the quality of evidence [31].

Above all, dietary modifications are necessary during orthodontic treatment. Patients must be motivated towards healthier eating habits. A study was designed with 180 patients to compare dietary intake between orthodontic patients and control subjects. The authors concluded that orthodontic patients consumed higher total calories, proteins, and carbohydrates (*p*>0.05) and significantly lower fiber, chromium, and beta-carotene (*p*<0.05) compared with the control group [32]. Riordan reported the effect of orthodontic treatment on dietary intake. A total of 10 adolescent patients were included in the study. The aim was to compare nutrient intake before and after orthodontic treatment. Recorded diets were analyzed using a two-sample t-test with an alpha level of 0.05. The results showed a decrease in copper and magnesium intake after orthodontic adjustments [33]. A qualitative study was conducted on 10 adolescent patients (four males, six females) to assess the early effects of fixed orthodontic treatment on dietary intake and behavior. In conclusion, the dietary habits of patients were changed as a result of pain. Therefore, orthodontists must provide nutritional guidance to their patients including increasing consumption of soft diets to avoid pressure sensitivity [34]. However, both studies were limited by their small sample size. In conclusion, a braces-friendly diet must be recommended by all the orthodontists [35]. Moreover, general dentists should also significantly contribute to giving oral hygiene and nutritional guidance to patients undergoing orthodontic treatment.

A recent study conducted by Cozzani *et al.* [36], shows that telephone follow-up after orthodontic treatment might be an effective procedure to reduce the pain threshold of patients. A question arose on whether to implement this procedure in a busy orthodontic practice or not.

## CONCLUSION

Orthodontists must use their best professional judgment to assess the pain threshold level of each patient individually. Effective orthodontist-patient communication and targeted nutritional guidance may help in preventing pain and discomfort experienced by the patients to some extent. However, based on the available literature, analgesics remained the effective and routine methodology of pain management. Moreover, while prescribing analgesics, orthodontists must be aware of the pharmacological action as well as the pros and cons related to each drug. A maximum recommended dose should be taken into consideration for each patient. Patients with special conditions such as psychological pain or trigeminal neuralgia often require collaboration with their specialists and advanced care management. Nevertheless, pain management is a complex phenomenon. Therefore, further investigations combining different methods of orthodontic pain control with appropriate study designs and large sample sizes are required.
